# Systemically administered peptain-1 inhibits retinal ganglion cell death in animal models: implications for neuroprotection in glaucoma

**DOI:** 10.1038/s41420-019-0194-2

**Published:** 2019-07-04

**Authors:** Dorota L. Stankowska, Mi-Hyun Nam, Rooban B. Nahomi, Renuka M. Chaphalkar, Sandip K. Nandi, Rafal Fudala, Raghu R. Krishnamoorthy, Ram H. Nagaraj

**Affiliations:** 1 Department of Pharmacology and Neuroscience, North Texas Eye Research Institute, UNT Health Science Center, Fort Worth, TX 76107 USA; 2 Sue Anschutz-Rodgers Eye Center and Department of Ophthalmology, University of Colorado School of Medicine, Aurora, CO 80045 USA; 3 Department of Microbiology, Immunology and Genetics, UNT Health Science Center, Fort Worth, TX 76107 USA; 4 Skaggs School of Pharmacy and Pharmaceutical Sciences, University of Colorado, Aurora, CO 80045 USA

## Abstract

Axonal degeneration and death of retinal ganglion cells (RGCs) are the primary causes of vision loss in glaucoma. In this study, we evaluated the efficacy of a peptide (peptain-1) that exhibits robust chaperone and anti-apoptotic activities against RGC loss in two rodent models and in cultured RGCs. In cultures of rat primary RGCs and in rat retinal explants peptain-1 significantly decreased hypoxia-induced RGC loss when compared to a scrambled peptide. Intraperitoneally (i.p.) injected peptain-1 (conjugated to a Cy7 fluorophore) was detected in the retina indicative of its ability to cross the blood-retinal barrier. Peptain-1 treatment inhibited RGC loss in the retina of mice subjected to ischemia/reperfusion (I/R) injury. A reduction in anterograde axonal transport was also ameliorated by peptain-1 treatment in the retina of I/R injured mice. Furthermore, i.p. injections of peptain-1 significantly reduced RGC death and axonal loss and partially restored retinal mitochondrial cytochrome c oxidase subunit 6b2 (COX 6b2) levels in rats subjected to five weeks of elevated intraocular pressure. We conclude that i.p. injected peptain-1 gains access to the retina and protects both RGC somas and axons against the injury caused by I/R and ocular hypertension. Based on these findings, peptain-1 has the potential to be developed as an efficacious neuroprotective agent for the treatment of glaucoma.

## Introduction

Glaucoma affects more than 60 million people worldwide, and nearly 8 million people have been blinded by this disease^1^. Approximately 3 million people in the U.S. have glaucoma, and this number is projected to increase to 6.3 million by 2050^2^. It is known that axonal degeneration and the death of retinal ganglion cells (RGCs) are the primary causes of vision loss in glaucoma^3–5^. However, the underlying mechanisms responsible for RGC death in glaucoma are not fully understood.

Small heat shock proteins (sHSPs) are a family of proteins that function as molecular chaperones and have anti-apoptotic properties. All members of the sHSP family contain an “α-crystallin domain” flanked by a hydrophilic C-terminus and a variable N-terminus. sHSPs inhibit protein denaturation and aggregation by binding to structurally perturbed proteins in an ATP-independent manner^6,7^. They also inhibit apoptosis by blocking several key steps in both the extrinsic and intrinsic pathways of apoptosis^8^. Hsp20, Hsp27, αA-crystallin and αB-crystallin are the major sHSPs in humans^9^. αA-crystallin is abundant in the lens, while the other three sHSPs are expressed in many other tissues^10^.

Previous reports have shown that αB-crystallin is present in the retina in photoreceptors, RPE cells, the inner nuclear layer (INL), nerve fiber layer (NFL), and RGCs^11–14^. An increase in intraocular pressure (IOP) at an early stage of the glaucomatous process has been found to drastically suppress both the mRNA and protein levels of α-crystallin in RGCs^15,16^. Ahmed et al^17^. also found a decrease in the mRNA expression of αA, αB, and βB2-crystallins in the retina of Brown Norway rats subjected to IOP elevation. Gene microarray analysis has also showed profound downregulation of αB-crystallin in the IOP elevated retina of rats^18^. Recent work by Park et al. demonstrated that, in a microbead model of IOP elevation in mice, some of the most prominently downregulated genes are members of the crystallin family and include αB-crystallin^19^. In DBA/2 J mice, the expression of nine crystallin genes are downregulated upon IOP elevation^20^, the level of crystallins in this model are found to initially decline after IOP elevation and subsequently recover to normal levels, possibly as a protective response. The downregulation of αB-crystallin has also been observed in the trabecular meshwork of IOP-elevated perfused glaucomatous human eyes^21^. Moreover, several studies have demonstrated the neuroprotective properties of full-length crystallins following IOP elevation^22–25^ or axonal injury in animal models^26–29^. Similarly, the intravitreal delivery of αB-crystallin is protective to RGCs during ischemia/reperfusion (I/R) injury in the rat retina^30^. Together, these observations imply that crystallins are essential for RGC survival, particularly under conditions of elevated IOP.

Sharma and colleagues have discovered that a short 21 amino acid core peptide within the “α-crystallin domain” of αB-crystallin (^73^DRFSVNLDVKHFSPEELKVKV^93^) has the ability, like its parent protein αB-crystallin, function as a molecular chaperone^31^. We have previously shown that the intraperitoneal (i.p.) injections of this peptide block lens epithelial cell apoptosis and cataract formation in sodium selenite-treated rat pups and also reduce stress-induced apoptosis in cultured cells^32,33^. We have now tested this peptide (we refer to the peptide as peptain-1 in this study) against RGC death in two animal models. Our data show that peptain-1 is cell permeable, that it can access the eye when injected i.p. and that it can inhibit RGC death, axonal transport impairment and a mitochondrial deficit in RGCs.

## Results

### αB-crystallin levels are reduced in human glaucomatous retinas

**Fig. 1:**
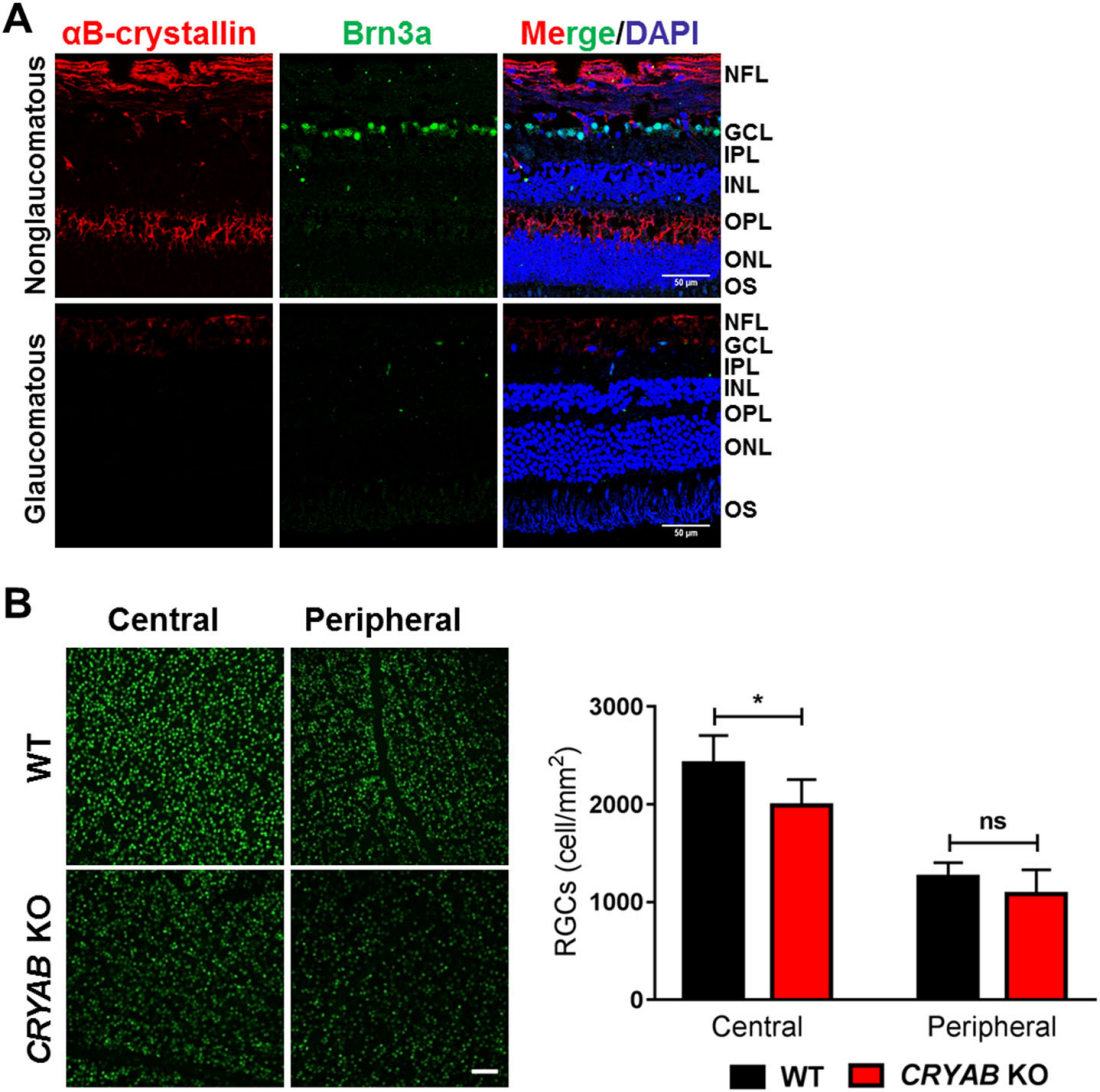
αB-Crystallin levels are lower in human glaucomatous retinas, and RGC counts are lower in *CRYAB* knockout (KO) mouse retinas. **a** αB-Crystallin (red) was detected in the nerve fiber layer (NFL) and in the outer plexiform layer (OPL) in the non-glaucomatous retinas. Only very weak immunostaining in the NFL was observed in the glaucomatous retinas. Representative images from the human retinas. The RGCs were labeled with a Brn3a antibody (green). The cell nuclei were stained with DAPI (blue). Scale bar = 50 μm. **b** The number of RGCs in the retinas of the *CRYAB* KO mice was lower than that in the retinas of the age-matched WT mice. The bar graphs represent the means ± SD of triplicate measurements. Scale bar = 200 μm. *ns* not significant, *p < 0.05 (unpaired *t*-test). GLC = ganglion cell layer

Previous studies have shown that there is a substantial decrease in crystallin levels after axotomy^34^ and also during steroid-induced ocular hypertension in rodents^35^. However, the status of αB-crystallin in human glaucomatous retinas is not clear. To address this, we performed immunostaining for αB-crystallin and Brn3a (to label RGCs) in retinal sections of glaucomatous human donor eyes and non-glaucomatous retinas from age-matched donors (Fig. 1a). We found substantial immunostaining for αB-crystallin in the NFL and outer plexiform layer (OPL) of the retinas of the non-glaucomatous eyes. Interestingly, in the retinas of the glaucomatous eyes, there was a drastic reduction in αB-crystallin in both the NFL and OPL. We also noted a loss of Brn3a positive cells in glaucomatous retinas, which corroborates several other previous studies that observed a decrease in the Brn3a and Brn3b proteins in animal models of glaucoma^36–39^.

### RGC numbers are lower in the retinas of αB-crystallin knockout (*CRYAB* KO) mice

To address if αB-crystallin has a constitutive role in maintaining the viability of RGCs, we counted the number of RGCs in wild type and *CRYAB* KO mice. The number of Brn3a-positive RGCs in *CRYAB* KO mice was significantly lower in the central (18%, *p* < 0.05) and slightly lower in the peripheral (16%) retinas, respectively, than in WT mice (Fig. 1b).

### Peptain-1 permeates rat RGCs

**Fig. 2:**
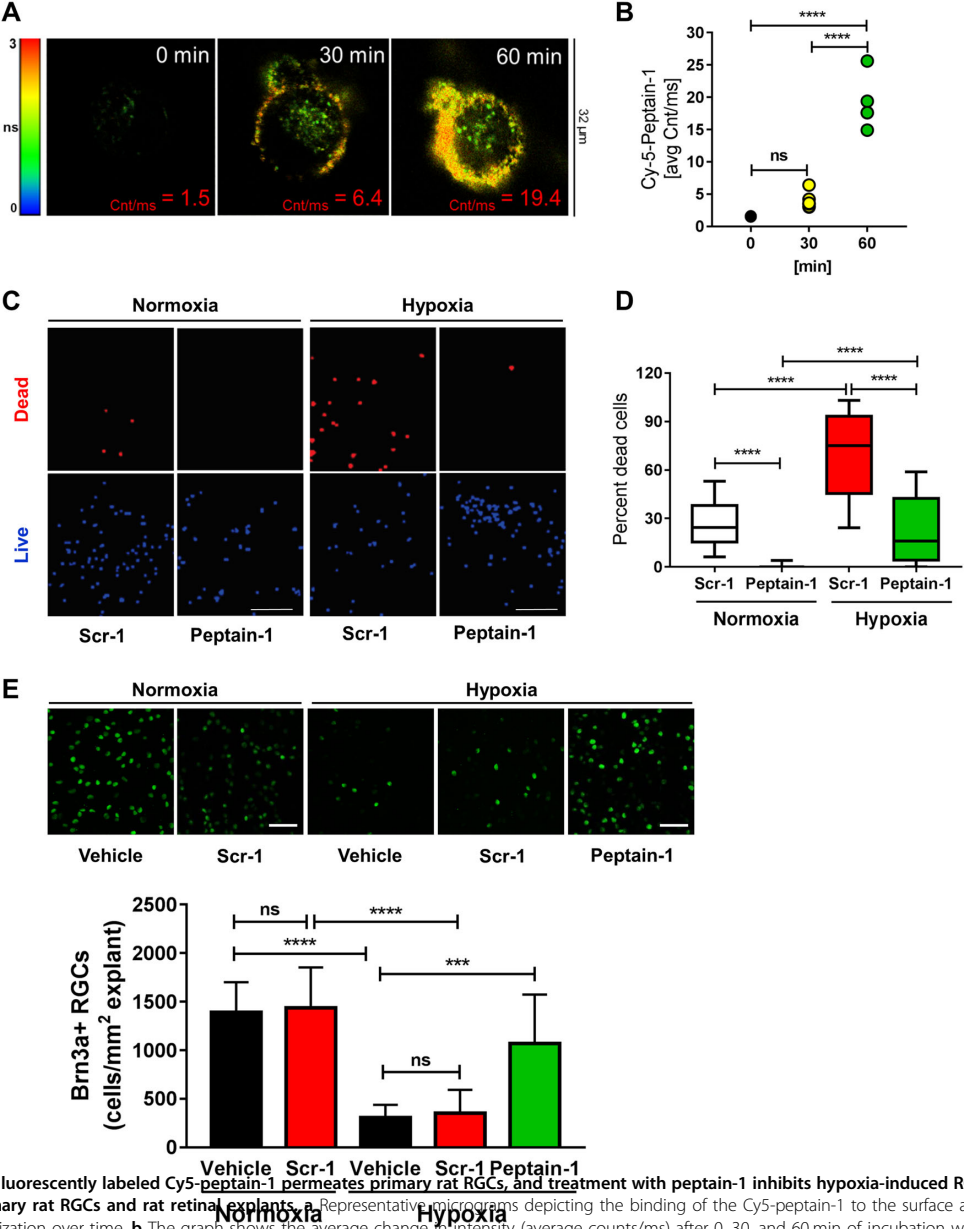
Fluorescently labeled Cy5-peptain-1 permeates primary rat RGCs, and treatment with peptain-1 inhibits hypoxia-induced RGC loss in primary rat RGCs and rat retinal explants. **a** Representative micrograms depicting the binding of the Cy5-peptain-1 to the surface and its internalization over time. **b** The graph shows the average change in intensity (average counts/ms) after 0, 30, and 60 min of incubation with Cy5-peptain-1 based on the changes shown in Fig. 2a. ns = not significant, ****p < 0.0001 (Tukey’s multiple comparison test). **c** and **d** peptain-1 protects primary rat RGCs from hypoxia-induced death. Primary RGCs were maintained either in a normoxic environment (10% CO_2_, 17.2% O_2_ at 37 °C) or in a hypoxic environment (10% CO_2_ and 0.5% O_2_ at 37 °C). The cells were treated with either 12.5 *μ*g/ml peptain-1 or 12.5 μg/ml scrambled peptide (Scr-1). ****p < 0.0001 (Tukey’s multiple comparison test). *n* = 4 per group in two independent experiments. E, Peptain-1 protects adult rat RGCs in retinal explants from hypoxic conditions. The retinal explants were immunostained with a Brn3a antibody (green) to detect for RGCs. The bottom panels show the quantification of RGCs per mm^2^ of the explant. Vehicle alone or Scr-1 induced no significant changes in the number of RGCs, but peptain-1 protected the RGCs. *ns* not significant, ***p < 0.001; ****p < 0.0001 (Tukey’s multiple comparison test). *N* = 4 explants per treatment

We wanted to determine whether peptain-1 is able to protect cultured RGCs against hypoxic stress. To test this possibility, we first investigated whether peptain-1 permeates RGCs using a sensitive technique called fluorescence lifetime imaging (FLIM). We labeled peptain-1 with sulfo-cyanine5 maleimide (Cy5) and incubated primary RGCs with the Cy5-peptain-1 for 0, 30 and 60 min. In cellular imaging using fluorescence intensity, the contribution from light scattering and sample autofluorescence imparts background signal on all images. FLIM offers two major advantages, namely, the separation of fluorescence emission not only spectrally by wavelength or emission color but also temporally by fluorescence lifetime. As shown in Fig. 2a, b, there was a time-dependent accumulation of Cy5-peptain-1 within the RGCs, beginning at 30 min, with intense labeling observed at 60 min (avg. cnt/ms 1.5 at 0 min and 19.4 at 60 min).

### Peptain-1 protects RGCs against hypoxic stress

After ensuring that peptain-1 has the ability to permeate RGCs, we tested the neuroprotective ability of peptain-1 under conditions of hypoxic stress. Previous studies have shown that in IOP elevated rat retinas that hypoxia markers are elevated in the areas of injured axons and the elevated levels correlate with the axonal transport impairment^40^. We therefore used hypoxia as an insult and tested the ability of peptain-1 to protect RGCs during hypoxic conditions. In this experiment, primary RGCs were treated with either peptain-1 or a scrambled peptide (Scr-1, 12.5 µg/ml) and exposed for 16 h to either a normoxic (17.2% oxygen) or hypoxic (0.5% oxygen) environment. Using a dead/live assay, we found that, compared with the scrambled peptide, peptain-1 was able to significantly (*p* < 0.0001) prevent hypoxia-induced cell death (Fig. 2c, d).

### Peptain-1 protects RGCs against hypoxic stress in rat retinal explants

We also tested peptain-1 in the adult retinal explants. The explants were cultured in 0.5% or 17.2% oxygen for 16 h with or without peptain-1. The RGC number was significantly (*p* < 0.0001) reduced in explants cultured under hypoxia when compared with explants cultured under normoxia (305 ± 132 and 1392 ± 309/mm^2^, respectively, Fig. 2e). We found that peptain-1 at 12.5 μg/ml significantly (p < 0.001) protected RGCs against the hypoxic insult. The number of RGCs in peptain-1- and scrambled peptide-treated explants was 1071 ± 152 and 305 ± 46/mm^2^, respectively.

### Intraperitoneally injected peptain-1 crosses the blood-retinal barrier and reaches the retina

**Fig. 3:**
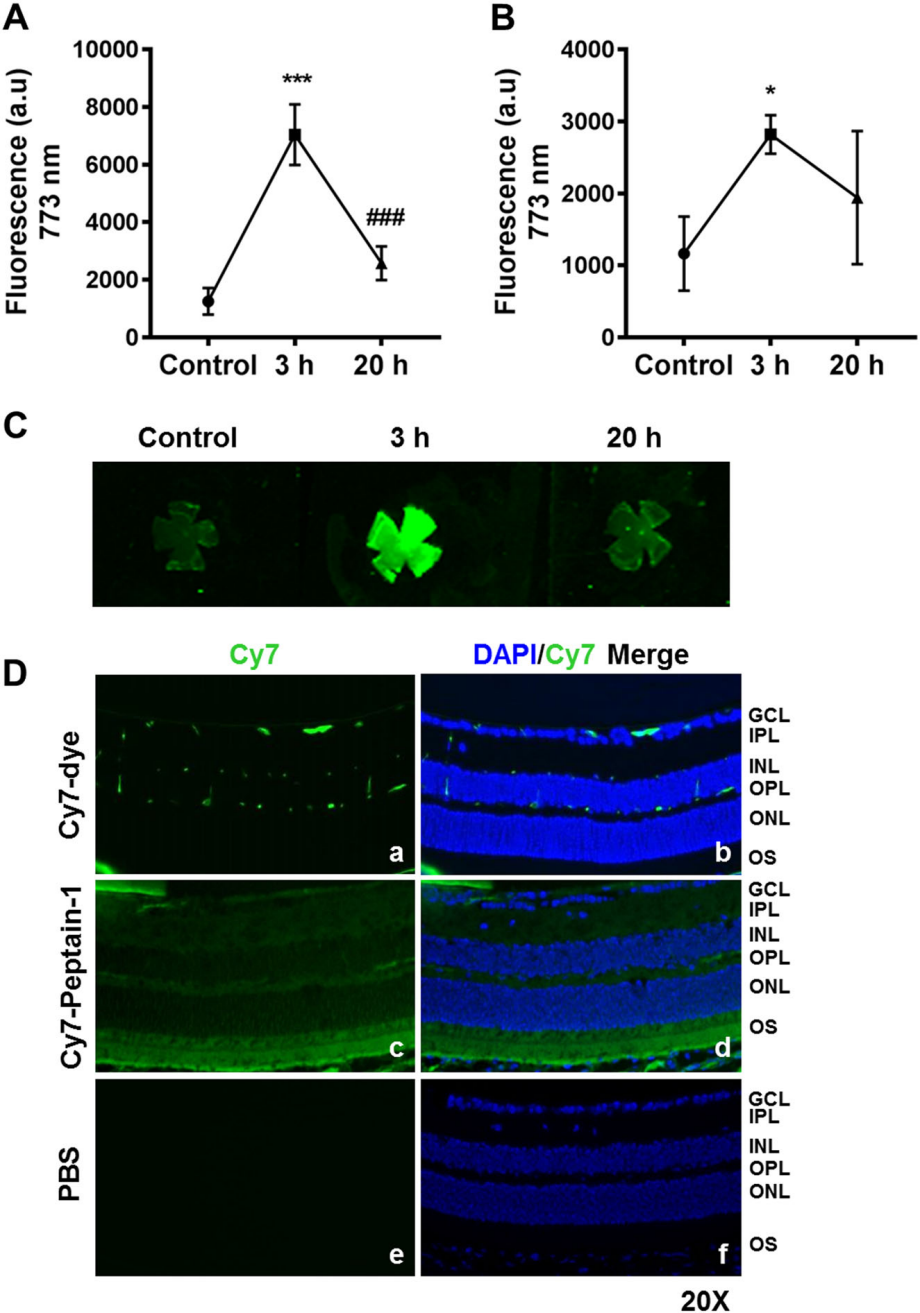
Detection of i.p.-injected Cy7-peptain-1 in the retina. The mice were sacrificed either 3 or 20 h after the i.p. injection of Cy7-peptain-1. Strong fluorescence, which peaked 3 h after injection, was detected in the serum (**a**) and retinal homogenates (**b**) in the Cy7-peptain-1 injected mice. Retina and serum of uninjected mice were used as controls. The line graph represents the means ± SD of triplicate measurements. *p < 0.05; ***p < 0.001 compared to the control, ^###^*p* < 0.001 compared to 3 h after injection (Tukey’s multiple comparison test). **c** Retinal flat mounts showed intense fluorescence 3 h after Cy7-peptain-1 injection, and the fluorescence decreased 20 h after injection. **d** Two hours after the i.p. injection of Cy7, the dye was detected in GCL, OPL, and possibly in the retinal blood vessels (**a**, **b**). The i.p. injection of Cy7-peptain-1 resulted in the detection of fluorescence in several layers of the retina, including the GCL, inner plexiform layer (IPL), OPL, inner segment (IS) and outer segment (OS) (**c**, **d**). A similar injection of PBS alone produced no fluorescence (**e**, **f**). Green = Cy7 dye. The cell nuclei were stained with DAPI (blue). Magnification = ×20

One of the main objectives of this study was to determine whether i.p. injected peptain-1 is able to prevent RGC loss in retinas subjected to I/R injury or elevation of IOP in rodents. To test this, we first investigated whether i.p. injected peptain-1 can be delivered to the retina. We labeled peptain-1 with a sulfo-cyanine7 maleimide (Cy7) fluorophore and administered Cy7-peptain-1 i.p. in mice. We found that peptain-1 levels peaked in the serum 3 h after injection and dropped to almost basal levels 20 h after injection (Fig. 3a). Cy7 fluorescence in the retinal homogenate showed a similar pattern (Fig. 3b). Similarly, in retinal flat mounts, peptain-1 levels peaked 3 h after injection and decreased 20 h after injection (Fig. 3c). To assess the distribution of peptain-1 in the retinal layers, the eyes were harvested 2 h post-injection, fixed and sectioned. We found that Cy7-peptain-1 was distributed throughout the retina, while in Cy7 alone injected mice, traces of Cy7 were observed in the inner retina (Fig. 3d). Fluorescence was not detected in the retina of mice injected with PBS alone, indicating that the observed fluorescence was specific to Cy7 and not due to autofluorescence artifacts. Together, these results suggest that i.p.-injected peptain-1 crosses the blood-retinal barrier and is distributed throughout the retina.

### Retinal αB-crystallin levels are reduced in the retina of mice subjected to I/R

**Fig. 4:**
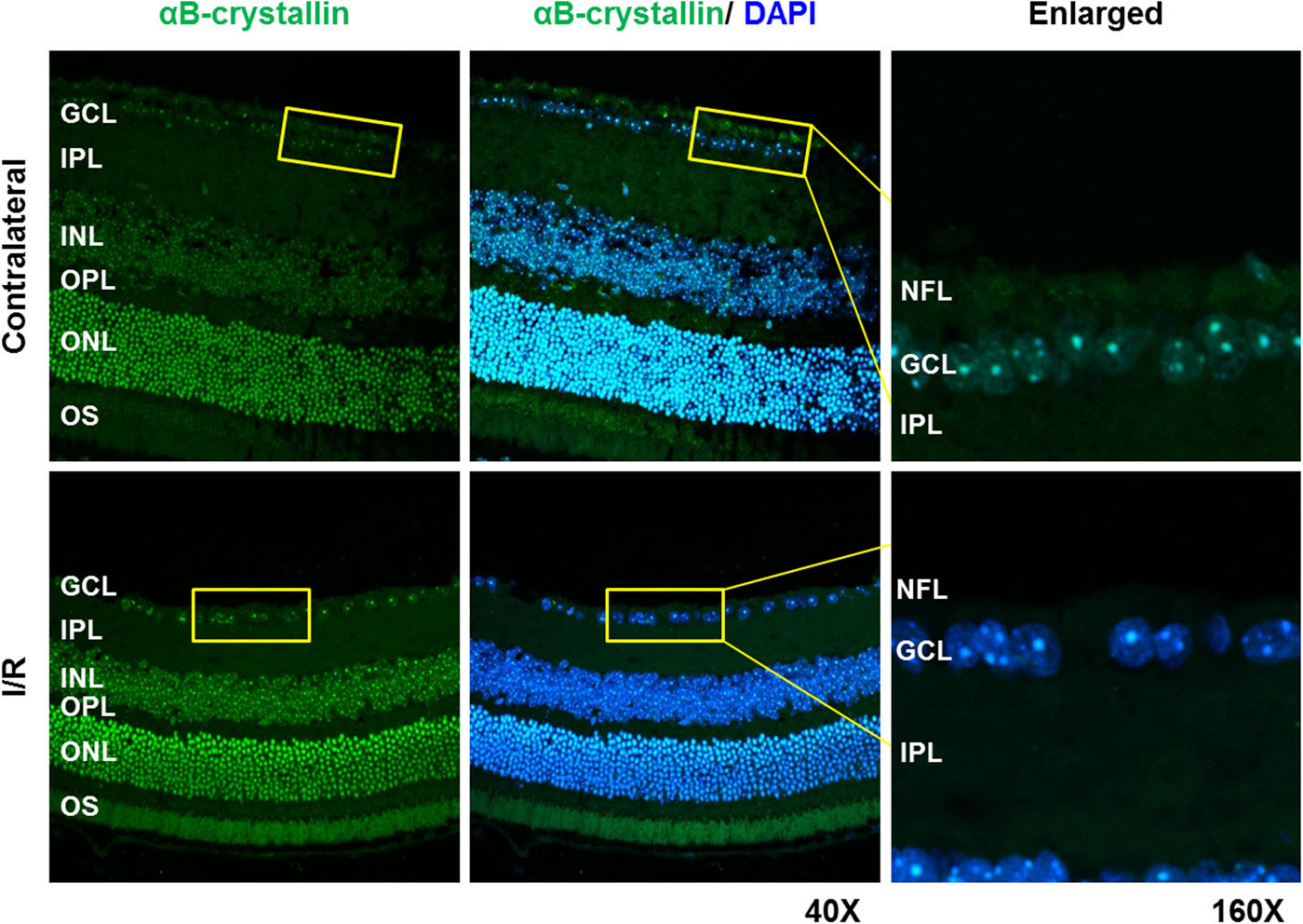
αB-crystallin levels are reduced in the retina of mice subjected to I/R injury. The morphological distribution of αB-crystallin in the retina of mice subjected to I/R injury. Fourteen days after I/R injury, the eyes of the mice were subjected to immunohistochemistry with an αB-crystallin antibody. The images shown are representative images from triplicate experiments. *N* = 3

WT mice were subjected to retinal I/R injury, and the retinal sections were immunostained for αB-crystallin. The αB-crystallin levels in the NFL and ganglion cell layer (GCL) were reduced in the I/R injured retinas compared to the uninjured contralateral retinas (Fig. 4), suggesting that the loss of αB-crystallin promotes RGC death under conditions of I/R stress.

### Peptain-1 administration inhibits RGC loss caused by I/R injury

**Fig. 5:**
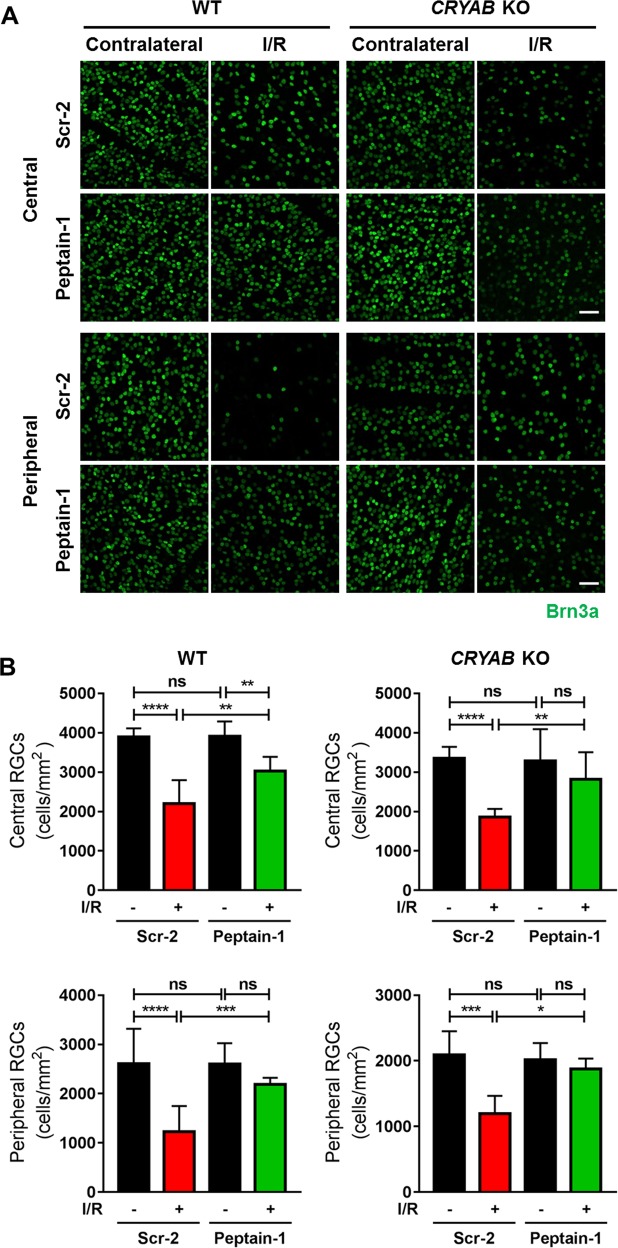
Inhibition of I/R-mediated RGC loss by peptain-1 in the mouse retina. **a** Fifty micrograms of a peptide (peptain-1 or Scr-2) was injected i.p. into either WT or *CRYAB* KO mice twice daily for 3 days following I/R injury. Fourteen days after injury, the number of Brn3a-positive RGCs (green) were counted in both the central and peripheral regions of the retina, and the numbers are shown in the bar graphs (**b**). *ns* not significant, *p < 0.05; **p < 0.01; ***p < 0.001; ****p < 0.0001 (Tukey’s multiple comparison test). *N* = 4. Scale bar = 50 μm

Fourteen days post-I/R injury, the retinas were dissected out and immunostained with a Brn3a antibody. In the WT mice injected with the scrambled peptide (Scr-2), the number of RGCs was significantly (*p* < 0.0001) decreased by 43 and 53% in the central and peripheral retinas, respectively (Fig. 5a, b). The administration of peptain-1 in the WT mice significantly inhibited RGC loss both in central and peripheral retinas (central: 23%, *p* < 0.01, peripheral: 16%, *p* < 0.001).

In the central retina of the *CRYAB* KO mice injected with the Scr-2, the number of RGCs was significantly lower compared to the WT injected with the Scr-2 (14% decrease, *p* < 0.001). Similarly, in the peripheral retina RGC counts were lower in the knockout animals than in the WT but not reaching statistical significance (20% decrease).

When the mice were subjected to I/R injury and injected with the scrambled peptide, the RGC number was significantly decreased by 45 and 42% in the central retina and peripheral retina, respectively (central: *p* < 0.0001 and peripheral: *p* < 0.001). However, there was no statistical difference in I/R mediated RGC loss between WT and *CRYAB* KO. The administration of peptain-1 in the *CRYAB* KO mice significantly inhibited RGC loss (central: *p* < 0.01, peripheral: *p* < 0.05); by 14 and 7% in the central and peripheral retina, respectively. Together, these results further suggest that peptain-1 is effective in inhibiting RGC loss induced by I/R injury, even in the absence of endogenous αB-crystallin.

### Peptain-1 inhibits the loss of axonal transport due to I/R injury

**Fig. 6:**
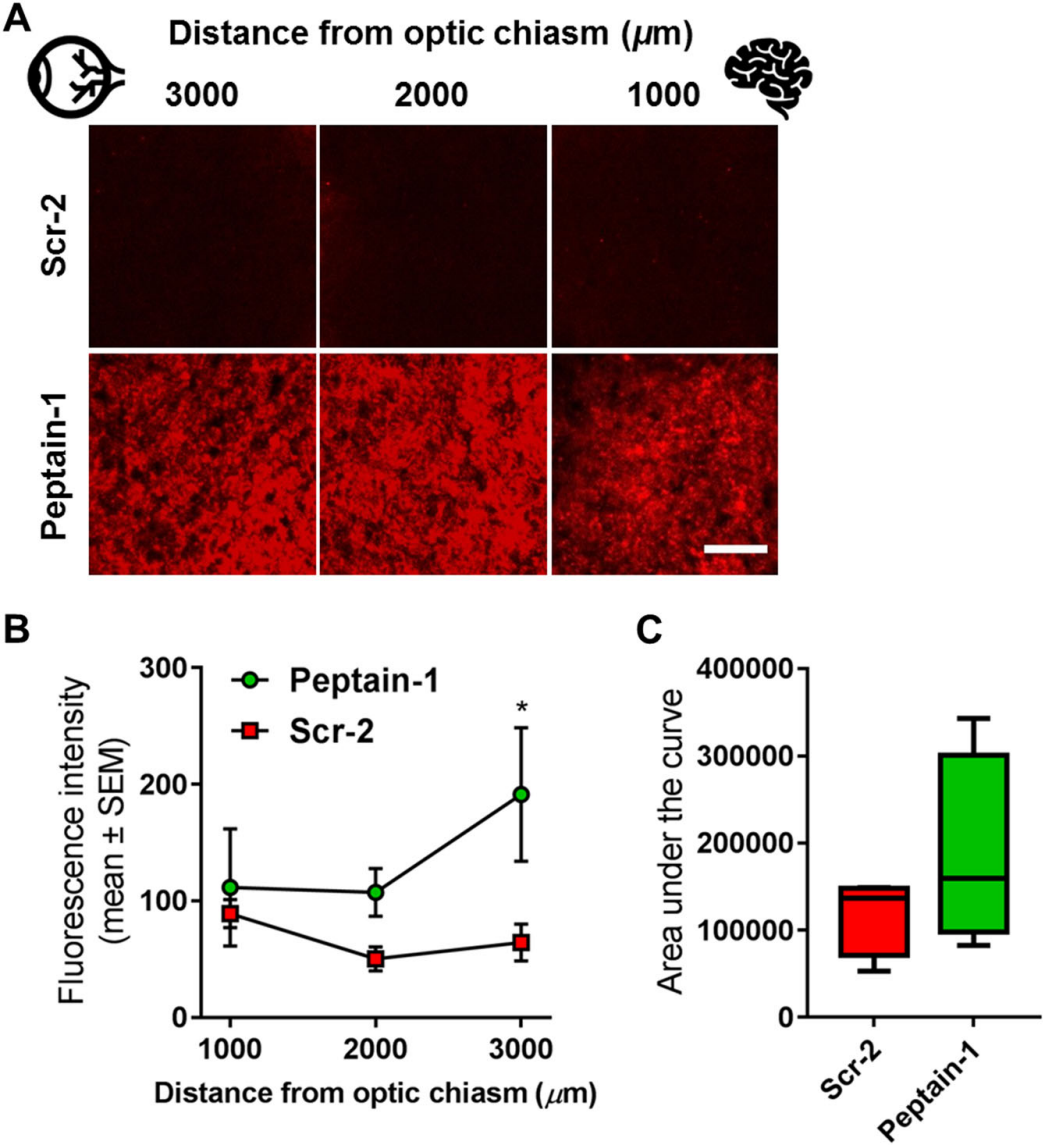
Defective axonal transport after I/R injury is partially corrected by peptain-1 administration. **a** Representative photomicrographs showing the optic nerve 3000, 2000 and 1000 μm from the optic nerve chiasm (indicated by the brain symbol) following I/R injury. The effect of 50 μg of Scr-2 (upper panels) or peptain-1 (lower panels) administered i.p. **b** The mean fluorescence intensity was measured across the width of each nerve at 1000-μm intervals along its length. The fluorescence intensity appeared lower in the Scr-2-treated animals 2000 and 3000 μm from the optic nerve chiasm when compared to peptain-treated animals. **c** The graph represents the differences in the areas under the curves for the peptain-1- and Scr-2-treated mice following I/R injury. The obtained values were also averaged for each group and compared using Kruskal-Wallis one-way analysis of variance on ranks. The data are plotted as the mean ± SEM. *N* = 4. *p < 0.05. Scale bar = 25 μm

While RGC counts provided an indication of their survival, their functionality was assessed by analyzing axonal transport. Axonal transport in the I/R-injured and cholera toxin subunit B-conjugated to Alexa Fluor 555 (CTB)-injected optic nerves was quantified by measuring the average fluorescence intensity across the width of the optic nerve at 1000-μm intervals from the optic chiasm along the full length of each optic nerve, as described by Bull et al^41^. The inhibition of axonal transport was clearly evident in the mice treated with Scr-2 (Fig. 6a), indicative of an amelioration of the axonal transport deficits. In particular, the CTB intensity closer to the eye (at 3000 µm distance from optic chiasm) was significantly higher (*p < 0.05) in peptain-1 injected eyes than in Scr-2 injected eyes (Fig. 6b). Statistical analysis of the area under the fluorescence intensity curve for each mouse was performed, and we found an approximately 40% increase (not statistically significant using a t-test) in the area under the fluorescence intensity curves between peptain-1 and Scr-2 (Fig. 6c).

### Peptain-1 inhibits RGC loss and protects against axonal injury in Morrison’s model of glaucoma in rats

**Fig. 7:**
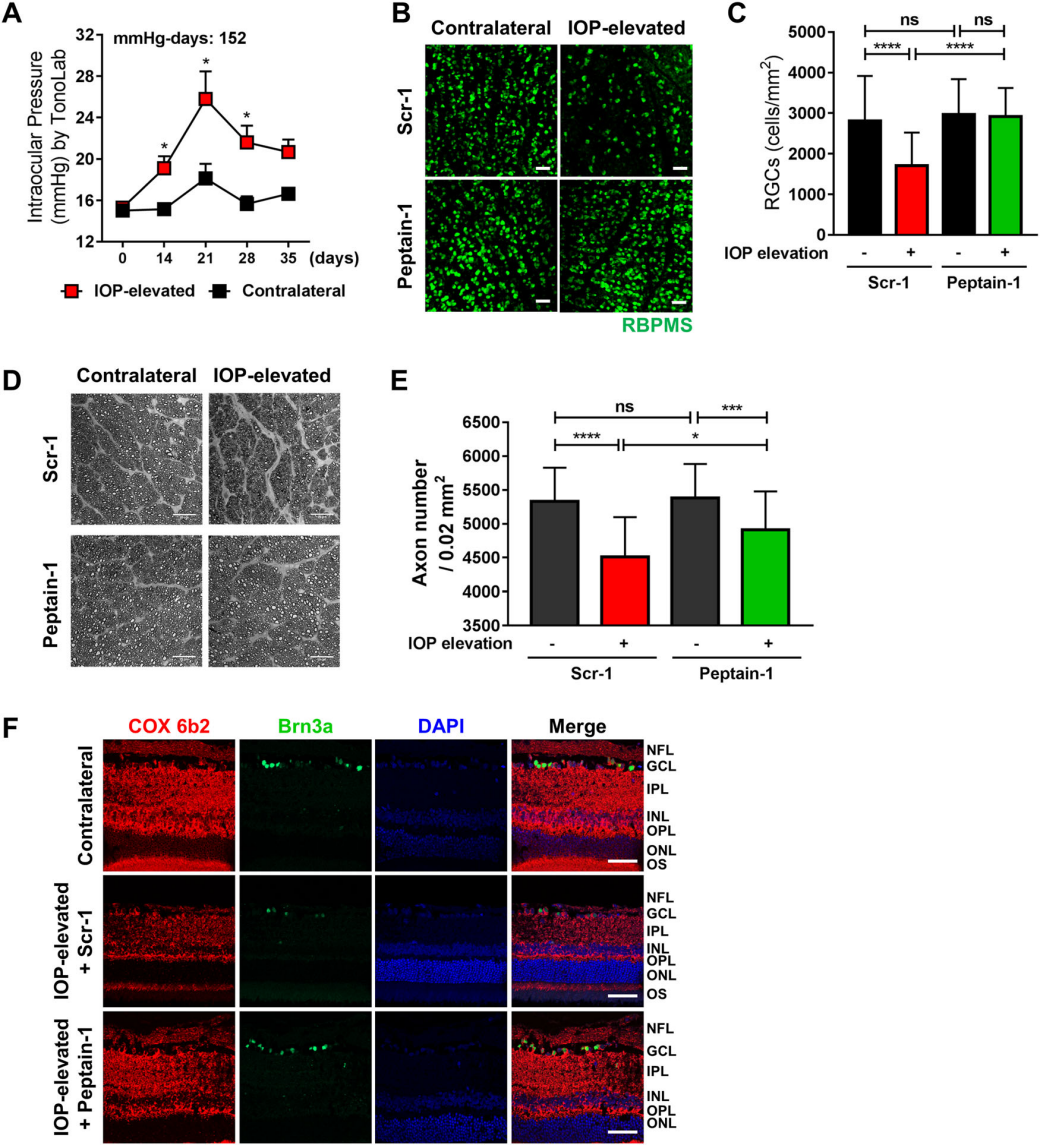
Peptain-1 inhibits RGC loss in Morrison’s rat model of ocular hypertension. Retired breeder Brown Norway rats (*n* = 3 per group) underwent IOP elevation and were subsequently injected i.p. with 10 μg of either peptain-1 or Scr-2 3 times a week. The sustained significant increase in IOP (*n* = 6) over a period of 5 weeks is shown in (**a**). *p < 0.03, *t*-test. The RGCs were immunolabeled with a RBPMS antibody (**b**) and counted (**c**). *ns* = not significant; ***p < 0.001; ****p < 0.0001 (Tukey’s multiple comparison test). **d** Staining of optic nerve axons in the eyes of the peptain-1 and Scr-1 treated rats following IOP elevation for 5 weeks (*n* = 2 per group, the scale bar represents 25 μm). **e** A box plot representing the number of axons. ns = not significant, *p < 0.05; ***p < 0.001; ****p < 0.0001 (Tukey’s multiple comparison test). **f** Peptain-1 inhibits COX 6b2 loss during IOP elevation in rats. Immunohistochemical analysis of COX 6b2 was carried out in the rat retinal sections, Scale bar = 50 μm

A sustained significant increase in the IOP (IOP exposure of 152 mmHg-days) was evident over a period of 5 weeks in the hypertonic saline-injected rat eyes (Fig. 7a). The control eyes exhibited an average IOP of 16 ± 0.58 mmHg over this period. After 5 weeks of IOP elevation (20 to 26 mmHg), the rats were euthanized, retinal flat mounts were prepared, and RBPMS-positive RGCs were counted. Compared with the administration of the scrambled peptide (Scr-1, 47% decrease), administration of peptain-1 significantly (only 2% loss, *p* < 0.0001) attenuated RGC loss (Fig. 7b, c). While a significant protection of RGC survival was observed in the peptain-1-injected rats compared with the Scr-1-administered rats, the axonal integrity in the optic nerve was assessed following PPD staining in these groups of animals. As shown in Fig. 7d, axonal integrity was compromised, and a loss of architecture of the axonal bundles was observed in the optic nerve sections from the IOP-elevated and Scr-1-treated rats. In contrast, the optic nerve sections from peptain-1-injected rats exhibited better preservation of axon morphology and improved axon counts (Fig. 7d, e). Our data clearly show that peptain-1 prevents RGC death and protects RGC axons from degeneration during IOP elevation.

### Mitoprotection by peptain-1

We investigated whether the protective effects of peptain-1 could be due to its beneficial effects on the mitochondria. We performed immunostaining for COX 6b2, a subunit of the cytochrome *c* oxidase complex (complex IV), which is the last enzyme in the mitochondrial electron transport chain. Our results showed that, compared to the administration of the Scr-1, the administration of peptain-1 partially restored the levels of COX 6b2 in the IOP-elevated rat retinas (Fig. 7f), suggesting that peptain-1 could be mitoprotective in RGCs.

## Discussion

Current treatments for glaucoma include eye drops and surgical manipulations to lower the IOP. However, neurodegeneration continues even when the IOP is lowered. While many key events of the pathology of glaucoma have been identified, the mechanisms underlying RGC death in glaucoma have not been fully elucidated. Although therapies aimed at preventing RGC death have shown success in animal models of glaucoma, further work is needed to identify drugs that can penetrate the retina in adequate amounts and specifically target RGCs.

In this study, we tested the neuroprotective properties of a core peptide of αB-crystallin (peptain-1, also referred in the literature as mini-αB or mini-αB chaperone). We demonstrated that systemic administration of peptain-1 prevents RGC loss. Our data indicate that peptain-1 blocks RGC death in vivo, inhibits the hypoxia-mediated apoptosis of RGCs in retinal explants and primary cultures and also protects against RGC axonal degeneration in vivo. Our study clearly showed that i.p.-injected peptain-1 is transported to the retina. However, it is unclear how peptain-1 is transported across the blood-retinal barrier. This transport could occur by passive diffusion or through mediation of a transporter. A previous study identified sodium-coupled oligopeptide transporters 1 and 2 (SOPT1, SOPT2) as possible mediators of the peptide transport into cells^42^. Whether these transporters play a role in the transport of peptain-1 to the retina needs to be further investigated.

The reduction in αB-crystallin levels in the glaucomatous human retinas suggests a direct link between αB-crystallin and the neurodegeneration of RGCs. Another important finding in our study was that the number of RGCs in the retinas of *CRYAB* KO mice was significantly lower than that in the retinas of WT mice. These results further emphasize the constitutive, pro-survival constitutive role of αB-crystallin for RGCs.

The neuroprotective ability of peptain-1 is fairly similar to that of full-length αB-crystallin. Previous studies have demonstrated the neuroprotective role of full length αB-crystallin in various animal models of glaucoma. For example, the intravitreal injection of αB-crystallin has been shown to be neuroprotective following optic nerve transection in rats^43^, and promote axonal regeneration after optic nerve crush in rats^44^. Additionally, the intravitreal delivery of αB-crystallin induces increased survival of RGCs after optic nerve axotomy in mice^34^ and in a rat model of hypertensive glaucoma^22^. Similarly, the intravitreal delivery of αB-crystallin has been found to be protective to the retina of rats during ischemia/reperfusion (I/R) injury^30^. In addition, intravenous injection of the full length αB-crystallin, similar to peptain-1, protects both RGC somas and axons following optic nerve crush in rats^27,45^.

Several previous studies have used peptides derived from α-crystallin as therapeutics. A peptide derived from αA-crystallin (mini-αA) conjugated to a cell penetrating signal peptide has been successfully used to prevent β-amyloid fibril formation and suppress β-amyloid toxicity in ARPE-19 cells^46^. Another study demonstrated retinal protection conferred by mini-αA in NaIO_3_-induced retinal degeneration in mice^47^. Mini-αB fused to an elastin polypeptide prevented retinal pigment epithelial cell atrophy and progressive retinal degeneration in a mouse model of age-related macular degeneration^48^. We have used αA- and αB-crystallin peptides (including peptain-1) to block selenite-induced cataracts in rats^32^. Altogether, these reports support the idea that peptain-1 is useful for retinal neuroprotection in vivo.

It is remarkable that peptain-1 delivered systemically is potent against two very different modalities of RGC death in vivo, ocular hypertension in rats and I/R injury in mice. To the best of our knowledge, there have been no other reports of a systemically delivered peptide being effective against RGC death. What is even more striking is that peptain-1, has the ability to prevent axonal transport deficits in the I/R model and reduce axonal damage in an ocular hypertensive rat model. Exogenously administered αB-crystallin has been shown to limit neuronal damage in animal models. Klopstein et al^49^. demonstrated that intravenously delivered αB-crystallin promotes locomotor recovery following spinal cord contusion in mice. αB-Crystallin is thought to interact and stabilize cytoskeletal proteins, leading to the prevention of cell death^50^. Furthermore, αB-crystallin has been shown to interact with desmin filaments and their aggregates^51^. The delivery of αB-crystallin in mice enhances remyelination and functional recovery after sciatic nerve injury by promoting the remyelination of regenerated peripheral axons^52^. Additionally, αB-crystallin has been shown to interact with neurofilament light chains^53^, one of the major components of the neuronal cytoskeleton, which is likely to provide stability to mature axons and regulate the axonal transport rate^54,55^. Whether peptain-1 possesses similar beneficial properties in the central nervous system for defective axonal transport needs to be further investigated.

It is known that mitochondrial abnormalities occur when defective αB-crystallin is expressed in tissues^56,57^. There is evidence for the expression of αB-crystallin in the mitochondria^57,58^, however, its role in this organelle is not completely understood. Based on our findings that the loss of COX 6b2 is alleviated by peptain-1, we propose that peptain-1 may limit mitochondrial damage in stressed RGCs. Further studies are needed to understand the molecular mechanisms for such protection.

The observed beneficial effects of peptain-1 could be also due to due to additional mechanisms including, its ability to bind metal ions, thereby limit oxidative damage^59,60^ and its anti-apoptotic activity, as demonstrated in previous studies^32,33^. Whether peptain-1 retains some other cell protective properties of αB-crystallin is not known. If it does, it could mimic αB-crystallin’s protective ability against H_2_O_2_-mediated apoptosis in cells^42,61^, loss of glutathione^32^ and the ability to block activation of microglia^62^ thereby restrict oxidative and inflammatory damage and prevent RGC death. In fact, inflammation and oxidative damage are integral to RGC loss by I/R injury^63,64^. Furthermore, αB-crystallin has been shown to bind to inflammatory cytokines^65^, and if such binding and sequestration occurs with peptain-1, it could provide another mechanism by which peptain-1 blocks RGC death.

A unique aspect of our study is that we administered peptain-1 i.p., while many other studies that have tested therapies to prevent RGC death by injecting them into the eye. We realize that the i.p. route employed in our study was to test the notion that peptain-1 is neuroprotective and is not a realistic method of its delivery for neuroprotection in glaucoma. For long-term glaucoma treatment, the drug should be easily administered, preferably through an ocular route, and more importantly, it should have an extended period of bioavailability after its delivery. In general, drugs applied topically, such as eye drops, have poor penetrance, with <1% reaching the retina. Moreover, repeated injections of drugs into the vitreous could result in retinal detachment, hemorrhage, endophthalmitis or posterior uveitis. In contrast to existing approaches using full-length α-crystallins, our future goal is to develop an efficacious delivery method for the sustained release of peptain-1 into the retina for therapeutic treatment against glaucoma. Along these lines, the stability of peptain-1 may be improved by acetylating lysine residues. Our previous study showed that acetylated peptain-1 is better than the unmodified peptide in preventing cataract development and lens epithelial cell apoptosis^33^. Furthermore, peptain-1 may also be used as an adjunct therapy along with drugs that lower the IOP to treat glaucoma.

## Conclusions

Systemically administered peptain-1 prevents RGC loss and confers protection against axonal degeneration and defective transport in rodent models of glaucoma. Our data show that peptain-1 permeates the blood-retinal barrier and partially restores the level of a mitochondrial electron transport chain complex protein. Moreover, peptain-1 significantly inhibits RGC loss even under acute I/R injury to the retina. We therefore propose that peptain-1 could potentially be developed as a first line of treatment to promote the neuroprotection of RGCs in glaucoma and in conditions of acute angle closure glaucoma, retinal vein or artery occlusion to prevent RGC death.

## Materials and methods

### Peptides

Peptain-1 (DRFSVNLDVKHFSPEELKVKV) and scrambled peptides, FEPSVRFSKVDHLVKENDLVK (Scr-1) and DRASVNLDVKHFSPEELKVKV^31^ (Scr-2), all >95% pure, were obtained from Peptide 2.0 (Chantilly, VA). All peptides used in this study were confirmed by mass spectrometry to have the expected molecular weight. The scrambled peptides, Scr-1 and Scr-2, were deemed nonfunctional based on their almost complete lack of chaperone activity against three client proteins (Fig. S1) and inability to protect RGC death from the retinal I/R injury in mice (Fig. S2).

### αB-Crystallin in human retinas

Paraffin-embedded retinal sections from two glaucomatous human donor eyes (from donors aged 74 and 76 years) and two retinas from non-glaucomatous eyes (from donors aged 62 and 79 years) were kindly provided by Dr. Abbott Clark, UNTHSC. Immunostaining for αB-crystallin (Cat# 1D11C6E6, Thermo Fisher Sci., Waltham, MA, 1:200 dilution) and Brn3a (Cat# sc-31984, Santa Cruz, 1:500 dilution) was performed as previously described for paraffin-embedded tissue sections^37,66^.

### Animals

All animal procedures were performed in compliance with the ARVO Statement for the Use of Animals in Ophthalmic and Vision Research and approved by the Institutional Animal Care and Use Committee (IACUC). All rats were purchased from Charles River Laboratories (Wilmington, MA). Wild-type (WT, C57BL/6 J or 129/sv) mice were obtained from Jackson Laboratories (Bar Harbor, ME, USA). *CRYAB* KO,129/sv were rederived from the original stock supplied by Dr. Eric Wawrousek from the National Eye Institute. Adult (10-week-old) female Sprague–Dawley rats were used for the retinal explant experiments. Male retired breeder Brown Norway rats (Rattus norvegicus; Charles River Laboratories, Wilmington, MA, USA) in the age group of 8–12 months were used in this study for IOP elevation.

### Permeability of peptain-1 conjugated to Cy5 in primary rat RGCs

Peptain-1 with an additional cysteine residue at the C-terminus (DRFSVNLDVKHFSPEELKVKVC, Peptide 2.0) was used for conjugation. Ten microliters of 0.5 N NaOH was added to 1 mg of peptain-1 in 1 ml of PBS to dissolve the peptide. To the dissolved peptide, 1 mg of Tris-(2-carboxyethyl)phosphine hydrochloride (TCEP, Thermo Fisher, Cat# 20490) was added, and the pH was adjusted to 7 with 0.5 N NaOH. This mixture was incubated at room temperature (RT) for 30 min. After incubation, 0.5 mg of Cy5 (Lumiprobe, Hunt Valley, MD, Cat# 23380) in 60 *μ*l of water was added, and the mixture was incubated initially at RT in a shaker for 2 h and then at 4 °C overnight. This mixture was dialyzed against PBS in a 1-kDa cutoff dialysis tube (Spectrum Lab, Irving, TX, Cat# 131084) overnight to remove the unbound dye.

The isolation of rat primary RGCs was performed according to a previously published protocol^37,66^. The purity of the culture was found to be between 90–95% in most preparations. The isolated cells were seeded in glass bottom dishes and cultured for 7 days as previously described^37,66^. The RGCs were then washed and maintained in phenol red-free DMEM without trophic factors and incubated for 0, 30 or 60 min with 0.25 µg/ml Cy5-peptain-1. To assess the penetration of Cy5-peptain-1 into the RGCs, we used an MT-200 (PicoQuant, Berlin, Germany) confocal microscopy system with a 60 × 1.2 NA Olympus water immersion objective and a 50 μm pinhole were used with an Olympus IX71 inverted microscope with a piezoelectric scanning stage (Physik Instrumente, Karlsruhe, Germany) for all fluorescence imaging measurements and lifetime measurements. A PDL 633 (633 nm wavelength) laser operating at a 20 MHz repetition rate by a PDL 828 “Sepia II” driver was used as the excitation source for all measurements, and 635 and 650 nm long pass filters (Semrock, NY, USA) were used to exclude the excitation signal from the detector. Symphotime V 4.2 (PicoQuant, Berlin, Germany) software was used to analyze and fit fluorescence lifetime decays.

### Peptain-1-mediated protection of RGCs against hypoxia in RGC cultures and retinal explants

Primary RGCs (isolated as above) were seeded on glass coverslips and exposed to either normoxic or hypoxic conditions in the presence of peptain-1 (12.5 µg/ml) or a scrambled peptide (12.5 µg/ml) for 16 h. RGC survival was assessed using a live/dead assay as previously published^67^. Rat retinal explants were prepared as previously published^68^. Four explants per group, each derived from a different animal, were cultured with the RGC layer facing up and maintained in humidified incubators with 5% CO_2_ at 35 °C (normoxia) for 16 h, as published previously^68^. Following one hour of equilibration under normoxic conditions, an ischemic chamber was used to culture the retinal explants in 0.5% O_2_ (hypoxia). Some retinal explants were treated with either peptain-1 (12.5 µg/ml) or a scrambled peptide (12.5 μg/ml) for 16 h under normoxic or hypoxic conditions. Retinal explants were fixed with 4% paraformaldehyde overnight at 4 °C and then permeabilized with PBS containing 0.1% sodium citrate and 0.2% Triton-X100 for 10 min. Blocking buffer (5% normal goat serum, 5% BSA in PBS) was applied to the explants for 24 h at 4 °C. After blocking buffer was removed and explants were incubated with goat anti-Brn3a (C-20) antibody (1∶500 dilution, Cat#. sc-31984, Santa Cruz Biotechnology, Dallas, TX) for 48 h at 4 °C. Explants were then washed three times in PBS for 5 min. Donkey anti goat Alexa 488 (Invitrogen, Cat# A11055, 1:1000 dilution) was added and incubated for 24 h in the dark at 4 °C. Explants were washed again three times with PBS and mounted and imaged using confocal microscope (Zeiss confocal laser scanning microscope LSM 510).

### Conjugation of peptain-1 with Cy7 and the detection of conjugated peptain-1 in the serum and retina of mice

Cy7 was conjugated to peptain-1 by a procedure similar to the one used to conjugate Cy5 to peptain-1 described above. The Cy7-conjugated peptide (250 μg peptide/animal), Cy7, or PBS was injected i.p. into the *CRYAB* KO mice. The animals were sacrificed 3 or 20 h after injection. Retina and serum of uninjected mice were used as controls. To obtain the serum, whole blood was collected and incubated at RT for 30 min prior to centrifugation at 2000x*g* for 10 min. The serum was diluted 1:100 in PBS, and the fluorescence was measured as described below. One retina per animal was dissected and fixed with 4% paraformaldehyde for 1 h at RT. Retinal flat mounts were prepared and transferred to microscope slides, and fluorescence images were collected using an Odyssey® CLx Imager (LI-COR Biotechnology, Lincoln, NE). The contralateral retina was homogenized in 150 μl of 1X RIPA buffer (Thermo, Cat# 89900) containing a protease inhibitor cocktail (1:100, Sigma) by a hand-held homogenizer. The homogenate was centrifuged at 14,000 × g for 10 min, and the supernatant was used for fluorescence measurement (excitation 755 nm and emission 850 nm) in a Fluromax-4 spectrofluorometer (HORIBA Scientific, Edison, NJ). The Cy7-peptain-1 or Cy7 or PBS alone injected mice were sacrificed after 2 h and the eyes were enucleated and fixed in Davidson’s fixative for 1 h at RT. After this, sequential incubation in sucrose gradient was carried out starting with 10% sucrose in PBS on ice. When the eyeball settled at the bottom of the tube, the medium was switched to 20% sucrose in PBS and incubated at 4 °C for overnight followed by incubation with 30% sucrose in PBS on ice until eyeball reached the bottom of the tube. The eyes were then embedded in Tissue-Tek optimal cutting temperature compound (Sakura Finetek USA, Torrance, CA) before sectioning in a cryostat. Sections were immersed in ice cold acetone and incubated at −20 °C for 10 min, followed by three washes with PBS. Nuclei were stained with DAPI and images were taken using a Zeiss Axio Observer 5 fluorescence microscope (Carl Zeiss Microscopy, Thornwood, NY).

### Induction of retinal ischemia-reperfusion injury (I/R) and peptain-1 treatment in mice

I/R injury was induced as previously described^64^ with slight modifications. Briefly, 12-week-old WT, or *CRYAB* KO mice were randomly divided into the following two groups: the group that received a scrambled peptide and the groups that received peptain-1. The mice were anesthetized with an i.p. injection of ketamine/xylazine, and anesthesia was confirmed by the lack of toe-pinch reflux. The mice were placed on a heating pad throughout the I/R injury procedure to maintain their body temperature. To perform the I/R injury, the right eye was first cannulated into the anterior chamber with a 33-gauge needle connected to an elevated pouch containing 250 ml of 0.9% NaCl solution. This resulted in an elevation of the intraocular pressure (IOP) to at least 120 mmHg, and the needle was removed after 40 min (in 129/sv) or 60 min (in C57BL/6 J) of this procedure. The animals were injected i.p. with 50 μg of peptain-1 or scrambled peptide in 100 μl of saline 3 h prior to and immediately after I/R, and twice per day for 2 days after the I/R injury. The contralateral eyes were used as additional controls. The animals were euthanized on day 14 post-I/R injury.

### αB-crystallin levels in I/R injured retinas

The eyes of the WT (129/sv) mice were subjected to I/R injury as described above and, after 14 days, were enucleated and fixed in Davidson’s fixative solution overnight followed by 10% normal buffered formalin for 2 h. The fixed eyes were dehydrated in 70% ethanol, paraffin-embedded and cross-sectioned (10 μm). The sections were blocked for 1 h with 5% normal donkey serum (Cat# 017-000-121, Jackson Immunoresearch Labs Inc., West Grove, PA) and incubated with an anti-αB-crystallin mouse monoclonal antibody (1:200 dilution, Developmental Studies Hybridoma Bank, University of Iowa, IA) overnight. After three washes with PBS, αB-crystallin was visualized by incubating the sections with an Alexa Fluor 488-conjugated donkey anti-mouse secondary antibody for 1 h at 37 °C.

### Quantification of RGCs in the mouse retina

The retinas were fixed in 4% paraformaldehyde (Cat# 15710, Electron Microscopy Sciences, Hatfield, PA) overnight. The retinas were then rinsed and permeabilized in PBS containing 0.1% sodium citrate and 0.2% Triton-X100 for 10 min and blocked with 5% normal donkey serum overnight at 4 °C. Next, the retinas were incubated with an RGC-specific Brn3a antibody (1∶500 dilution, Cat# sc-31984, Santa Cruz Biotechnology, Dallas, TX) for 72 h at 4 °C. After being washed three times with PBS, the retinas were incubated with an Alexa Fluor 488-conjugated donkey anti-goat IgG secondary antibody (Cat# A11055) overnight at 4 °C. After being washed five times with PBS, the retinas were cut into 4 petal shapes using a sharp scalpel blade to make a retinal flat mount. The number of Brn3a-positive RGCs in the central and peripheral regions of the four quadrants of the whole-mounted retina was imaged in the confocal microscope (Nikon Eclipse Ti, Nikon instruments Inc. Tokyo, Japan) and counted using the ImageJ software (NIH).

### Axonal transport in mouse retinas

Twelve-week-old WT (129/sv) mice were subjected to I/R injury in the right eye as described above. On day 13 after I/R, the mice were anesthetized with ketamine/xylazine and injected intravitreally with 2 μl of 0.2% Cholera Toxin Subunit B, Alexa Fluor™ 555 Conjugate (CTB) (Thermo Fisher, Cat# C22843). After the injection, the needle was slowly withdrawn, and the injected area was treated with an antibiotic ointment. After 24 h, the animals were sacrificed, optic nerves were collected and cryosectioned. Sections at various distances from the chiasm from both experimental groups were imaged by confocal microscopy (Nikon Eclipse Ti, Nikon instruments Inc. Tokyo, Japan) and mean fluorescence intensities were analyzed using the ImageJ software (NIH).

### Morrison’s model of ocular hypertension in rats

To elevate the IOP in one eye, 50 μl of 1.8 M NaCl was injected into the episcleral veins of anesthetized rats to blanch the aqueous plexus, as previously described^37,66^. IOP measurements were performed in the rats two times per week at baseline (prior to surgery) and continued on postsurgery day 7 using a TonoLab (Colonial Medical Supply, Windham, NH) following the topical corneal application of 0.1% proparacaine, according to a published method^37,66^. Ten micrograms of peptain-1 or a scrambled peptide (in 200 μl of normal saline) was i.p. injected into the IOP-elevated rats three times a week for 5 weeks. The contralateral eyes served as additional controls. Retinal flat mounts were prepared, and the RGCs were stained for RNA-binding protein with multiple splicing (RPBMS, GTX118619, GeneTex)) and counted as previously described^66,68^. Briefly, RGC survival was assessed by counting RBPMS labelled cells in two eccentricities (E1 located 1/3rd and E2 located 2/3rd distance from the optic nerve head) within each retinal quadrant (superior, inferior, nasal, and temporal). The Fig. 7a shows micrographs collected from eccentricity 2 and Fig. 7b represents cumulative counts of RGCs per mm^2^ of the retina from both eccentricities and all quadrants.

### Axonal counts of rat retinas

Optic nerve cross-sections were obtained from a total of four IOP-elevated rats administered peptain-1 or a scrambled peptide for 5 weeks. The optic nerves were fixed in 2% paraformaldehyde and 2.5% glutaraldehyde in 0.1 M sodium cacodylate buffer for 3 h at RT. After embedding the optic nerves in resin, optic nerve cross-sections were obtained and stained with 1% paraphenylenediamine. The slides were imaged on a Zeiss LSM 510 META confocal microscope at ×63 magnification. Semiautomated counting of the axons was carried out in a masked manner to obtain the total axon counts. The images were analyzed and processed using NIH ImageJ software. The images were adjusted for brightness and contrast followed by conversion to 8-bit grayscale images to obtain representative images. The threshold values were adjusted, and an ImageJ particle analyzer plugin was used to assess the total number of particles or axons. The size and circularity criteria were adjusted and set along with the specification of bare outlines. The areas identified by the software as non-axonal or glial masses by visual inspection were excluded from the analysis. Axon counts from five randomly selected fields within each optic nerve were obtained. The average axon density of each region was presented as the axon count/0.02 mm^2^. We used identical parameters of analyses across all images.

### Immunohistochemical detection of cytochrome c oxidase complex 6b2 (COX 6b2) in rat retinas

Retinal sections were deparaffinized in xylene for 5 min and then rehydrated in a series of ethanol washes (100, 95, 80, 70, 50%). Blocking was carried out with blocking buffer containing 5% donkey serum in PBS overnight at 4 °C. Primary antibodies goat-anti Brn3a, rabbit-anti COX 6b2 (1:200, Abcam Cat# AB134960) were applied and sections were incubated overnight at 4 °C. The appropriate secondary antibodies were used in a 1:1000 dilution and incubated for 3 h at RT. DAPI staining was used for nuclei. The sections were imaged using a Zeiss laser scanning confocal microscope LSM 510.

### Statistical analysis

GraphPad Prism software version 7 (GraphPad Prism Software, Inc., San Diego, CA) was used for statistical analyses. We used one-way ANOVA and multicomparison tests to determine the significance of the differences among the treatment groups. A *p* value of <0.05 was considered statistically significant. When two groups were compared, a *t*-test was used for the statistical analysis.

## Supplementary information

Supplemental Figs 1 and 2
