# Correction: Systemically administered peptain-1 inhibits retinal ganglion cell death in animal models: implications for neuroprotection in glaucoma

**DOI:** 10.1038/s41420-019-0201-7

**Published:** 2019-07-31

**Authors:** Dorota L. Stankowska, Mi-Hyun Nam, Rooban B. Nahomi, Renuka M. Chaphalkar, Sandip K. Nandi, Rafal Fudala, Raghu R. Krishnamoorthy, Ram H. Nagaraj

**Affiliations:** 1 Department of Pharmacology and Neuroscience, North Texas Eye Research Institute, UNT Health Science Center, Fort Worth, TX 76107 USA; 2 Sue Anschutz-Rodgers Eye Center and Department of Ophthalmology, University of Colorado School of Medicine, Aurora, CO 80045 USA; 3 Department of Microbiology, Immunology and Genetics, UNT Health Science Center, Fort Worth, TX 76107 USA; 4 Skaggs School of Pharmacy and Pharmaceutical Sciences, University of Colorado, Aurora, CO 80045 USA

**Correction to: Cell Death Discovery**

10.1038/s41420-019-0194-2 published online 4 July 2019

Following publication of the original article, the authors noticed that scale bars were missing in Fig. 7 and from the four labels in Fig. 2c. The corrected figures are shown below, and the PDF and HTML versions of the paper have been modified accordingly.

**Fig. 2:**
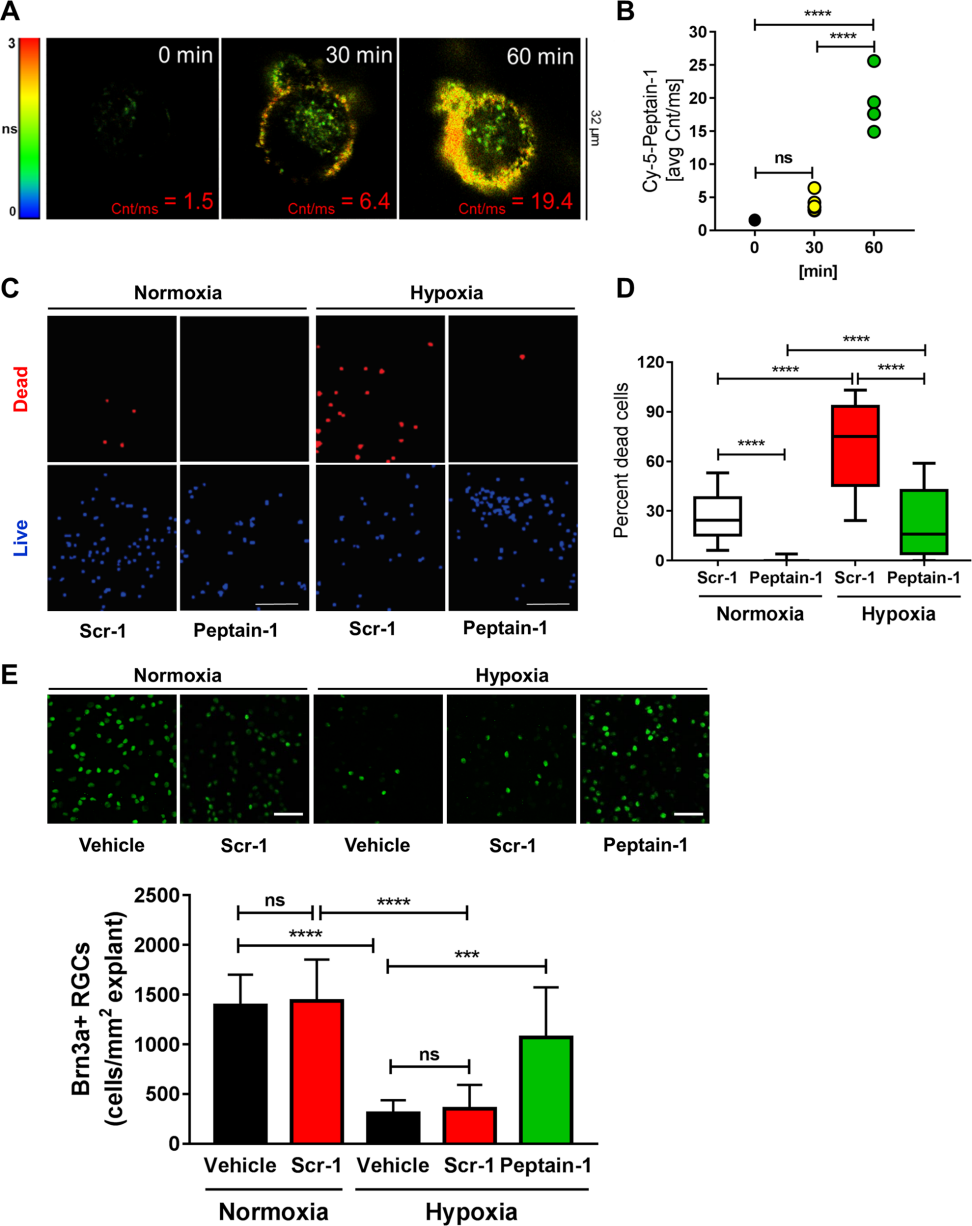
▓

**Fig. 7:**
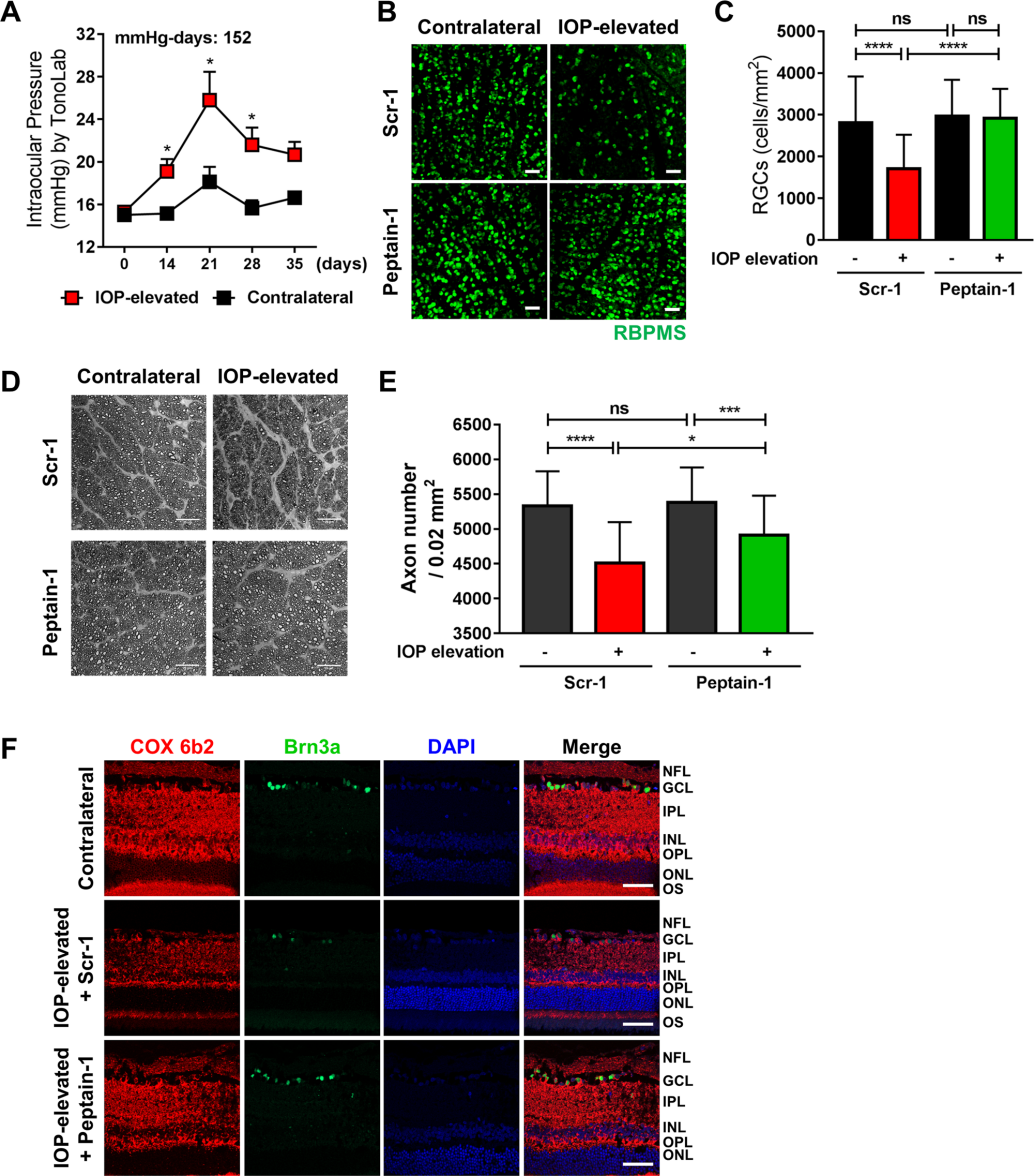
▓

The authors apologize to readers for the inconvenience.

