# Supplementary material for: Systemically administered peptain-1 inhibits retinal ganglion cell death in animal models: implications for neuroprotection in glaucoma
Source: Cell Death Discov. 2019 Jul 4;5:112. doi: 10.1038/s41420-019-0194-2 (PMC6609721; doi:10.1038/s41420-019-0194-2)
Supplement: Supplementary file 1 — Supplemental Figs 1 and 2 [file 41420_2019_194_MOESM1_ESM.docx]

**
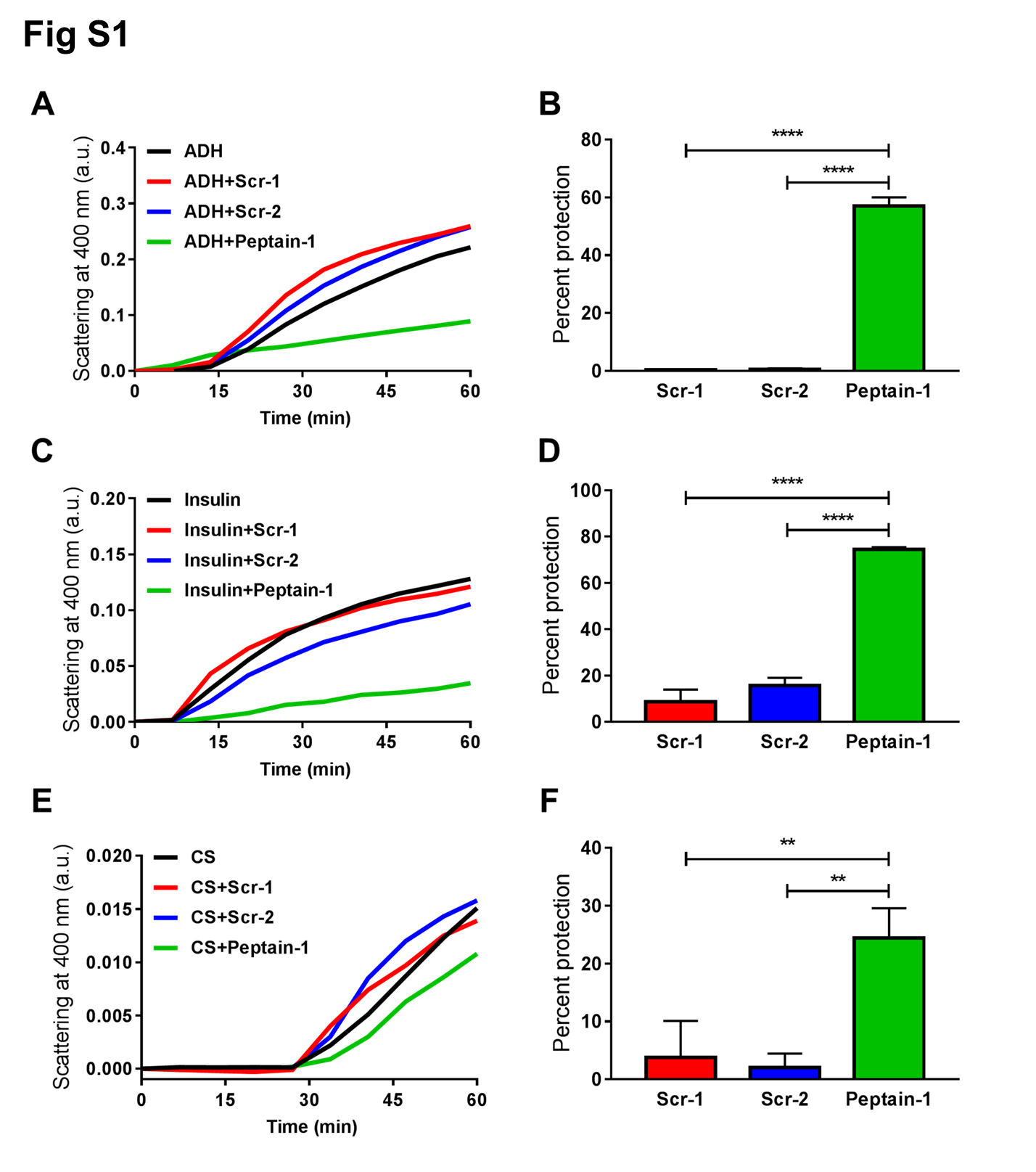
Figure S1. Scrambled peptides have negligible chaperone activity.** The chaperone activity of peptain-1, Scr-1 and Scr-2 was determined against (**A**) thermal aggregation of ADH at 43°C, (**C**) DTT induced aggregation of insulin at 25°C and (**E**) thermal aggregation of CS at 43°C. The ratio of peptide to client proteins used in these assays were 1:1 (W/W) for ADH and CS assay, 1:5 (W/W) for insulin assay. Chaperone activity assays were performed as previously described.^31, 69, 70^ The percent protection by peptides against aggregation of ADH, insulin and CS are shown in panel **B**, **D** and **E**, respectively. The bar graphs represent the means ± SD of triplicate measurements. **p < 0.01, ****p < 0.0001.

**
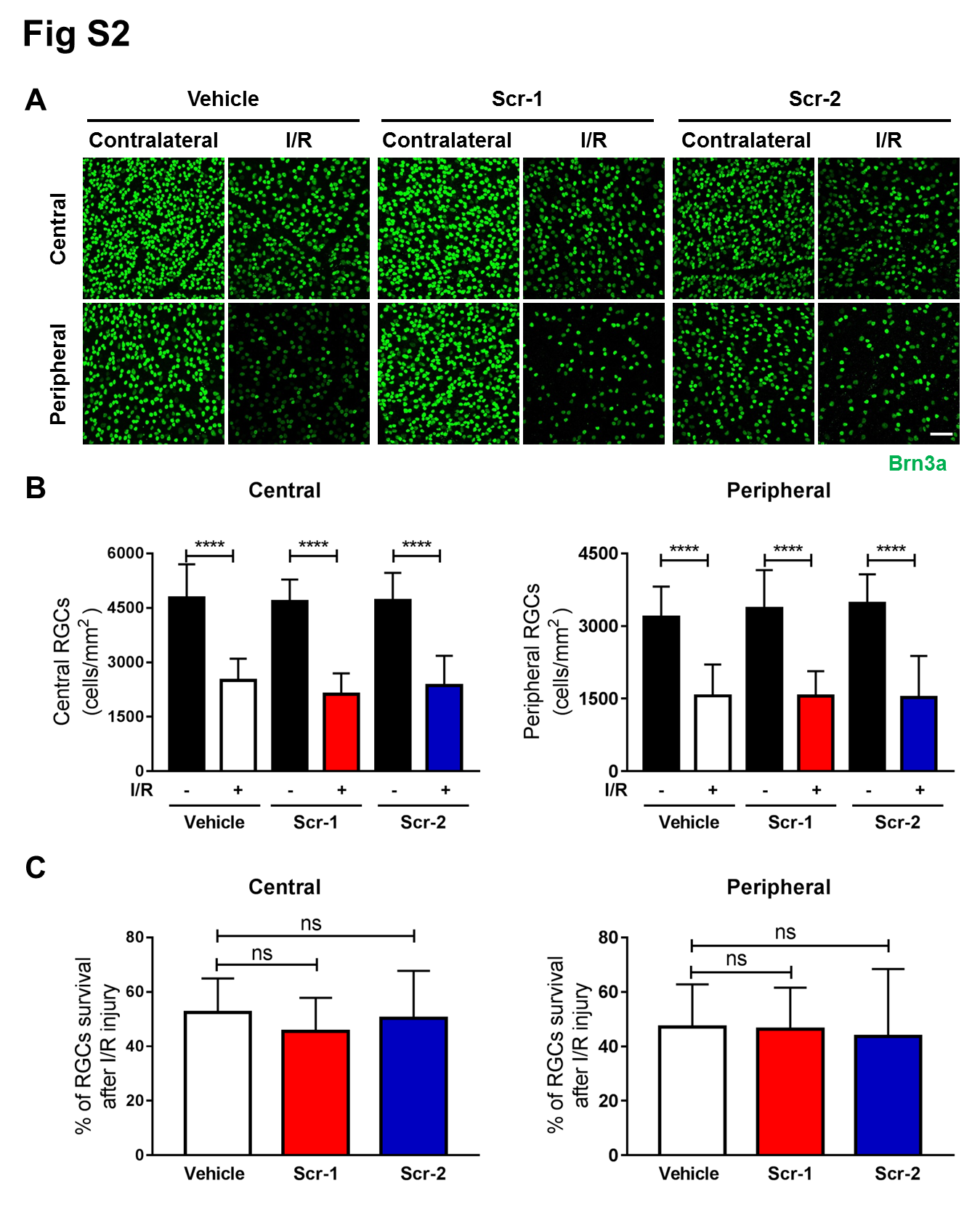
Figure S2. Scrambled peptides do not protect RGCs in I/R injured retinas of mice.** Fifty micrograms of a peptide (Scr-1 or Scr-2) was injected i.p. into WT (C57BL/6J) mice twice daily for 3 days following I/R injury. PBS containing 0.1% DMSO (100 *μ*l) was injected as a vehicle. Fourteen days after injury, the RGCs were immunostained with Brn3a antibody (green) (**A**) and the number of Brn3a-positive RGCs were counted in both the central and peripheral regions of the retina(**B**). Percentage of remaining RGCs after I/R injury are shown in the bar graphs (**C**). ns= not significant, ****p<0.0001 (Tukey's multiple comparisons test). N=3-4. Scale bar = 50 *μ*m.
